# Comparative Evaluation of Remineralizing Potential of Three Pediatric Dentifrices

**DOI:** 10.5005/jp-journals-10005-1361

**Published:** 2016-09-27

**Authors:** Ashna Kapoor, KR Indushekar, Bhavna G Saraf, Neha Sheoran, Divesh Sardana

**Affiliations:** 1 Postgraduate Student, Department of Pedodontics and Preventive Dentistry, Sudha Rustagi College of Dental Sciences and Research, Faridabad Haryana, India; 2 Professor and Head, Department of Pedodontics and Preventive Dentistry, Sudha Rustagi College of Dental Sciences and Research, Faridabad Haryana, India; 3 Professor, Department of Pedodontics and Preventive Dentistry, Sudha Rustagi College of Dental Sciences and Research, Faridabad Haryana, India; 4 Senior Lecturer, Department of Pedodontics and Preventive Dentistry, Sudha Rustagi College of Dental Sciences and Research, Faridabad Haryana, India; 5 Senior Resident, Department of Pedodontics and Preventive Dentistry, All India Institute of Medical Sciences, New Delhi, India

**Keywords:** Dentifrices, Fluoride, Remineraliation.

## Abstract

**Introduction:**

Dentifrices are available in different formulations and more commonly a single dentifrice is used by whole family; be it an adult or child. However, concerns over high fluoride in pediatric formulations coupled with inability of the children to spit have led to recommendations to minimize fluoride ingestion during toothbrushing by using a small amount of toothpaste by children and incorporating minimal quantity of fluoride in the toothpastes. Literature is scarce on the remineralization potential of popularly known Indian pediatric dentifrices; hence, pediatric dentifrices containing lesser concentration of fluoride have been marketed relatively recently for the benefit of children without posing a threat of chronic fluoride toxicity at the same time.

**Aim and objectives:**

The present study was undertaken to evaluate and compare the remineralization potential of three commercially available Indian pediatric dentifrices with different compositions on artificially induced carious lesions *in vitro* through scanning electron microscopy (SEM).

**Materials and methods:**

The present *in vitro* study was conducted on 45 sound extracted primary molar surfaces divided into three groups (15 each). Artificial demineralization was carried out, followed by remineralization using dentifrice slurry as per the group allocation. All the samples were studied for remineralization using SEM and the results statistically compared.

**Results:**

All three dentifrices tested showed remineralization; although insignificantly different from each other but significantly higher compared to the demineralizing surface.

**Conclusion:**

One can use pediatric dentifrices for preventing dental caries and decelerating lesion progression with an added advantage of lower fluoride toxicity risk.

**How to cite this article:**

Kapoor A, Indushekar KR, Saraf BG, Sheoran N, Sardana D. Comparative Evaluation of Remineralizing Potential of Three Pediatric Dentifrices. Int J Clin Pediatr Dent 2016;9(3):186-191.

## INTRODUCTION

Dental caries is a disease of the hard tissues of the teeth caused by interactions between cariogenic bacteria in dental plaque, fermentable carbohydrates (mainly sugars), and an imbalance in the process of demineralization and remineralization over time.^1^ When the oral environment favors demineralization, it leads to dissolution of hydroxyapatite and diffusion of calcium and phosphate ions toward the enamel surface - the process that can be reversed by remineralization. The clinical importance of remineralization was realized as early as 1966 by Backer Dirks, who found that nearly half of the white spot lesions in young individuals can remineralize.^2^ The remineralization capacity of incipient lesions under the influence of fluoride could be due to increased uptake of fluoride by surface and subsurface enamel in comparison to the adjacent sound enamel due to increased porosity and surface area of the deminer-alized area.^3,4^ Fluoride toothpastes and mouth rinses are the most widely used products to deliver fluoride to the oral cavity at an individual level; however, for children, only dentifrices are the most common source of fluoride ions.^5^ Dentifrices are available in different formulations and more commonly a single dentifrice is used by whole family; be it an adult or child. Pediatric dentifrices containing lesser concentration of fluoride have been marketed relatively recently for the benefit of children without posing the threat of fluoride toxicity at the same time. Literature is scarce on the reminer-alization potential of pediatric dentifrices; hence, the present study was undertaken to evaluate and compare the remineralization potential of three commercially available pediatric dentifrices on artificially induced carious lesions *in vitro* through scanning electron microscopy (SEM).

## MATERIALS AND METHODS

The present *in vitro* study was conducted on 45 sound extracted primary molar surfaces. The primary molar teeth that were collected were extracted due to orthodontic reasons. Presence of carious lesion, white spot, hypoplasia, discoluration, or crack line formed the exclusion criteria of the study. The teeth specimens were divided into three groups of 15 samples each as follows:

1. *Group A (Dentifrice 1):* Sodium fluoride 0.24% (0.15% w/v fluoride ion) 1000 ppm.
  *Composition:* Sorbitol, water, hydrated silica, sodium lauryl sulfate, PEG-32, flavor, cellulose gum, sodium fluoride, sodium saccharin, vitamin E, calcium gluconate, MICA/CI 77891, CI 19140.
2. *Group B (Dentifrice 2):* Sodium fluoride 500 ppm.
  *Composition:* Sorbitol, Water, Hydrated Silica, Sodium Lauryl Sulfate, PEG-32, Flavor, Cellulose Gum, Sodium Fluoride, Sodium Saccharin, Vitamin E, Calcium Gluconate, MIica/CI 77891, CI 12490.
3. *Group C (Dentifrice 3):* 498 ppm sodium MFP and xylitol.

### Demineralizing Procedure^6^ (Figs 1A to C)

The demineralizing solution was prepared with the help of the following components in equal proportions:

- 2.2 mM CaCl_2_
- 2.2 mM NaH_2_PO_4_
- 0.05 M acetic acid
- 1 M KOH
- Double-distilled water
- Hilndicator pH paper.

Freshly extracted sound primary molars were selected, cleaned, debrided, and sectioned with a safe-sided diamond disk to separate the crown and root portion. The crown was sectioned sagittally to obtain sound buccal enamel surface. Each specimen was then divided equally into three equal thirds [one-third positive control (sound surface), one-third negative control (demineralized), and the other third served as the study group (pediatric dentifrice)]. All the 45 specimens were suspended with the floss immersing their two-thirds of the surface in the demineralizing solution, while one-third of the surface was covered with nail varnish and served as positive control (sound surface). The specimens were suspended for 96 hours in the demineralizing solution before washing them with double-distilled water for 15 seconds.

**Figs 1A to C:**
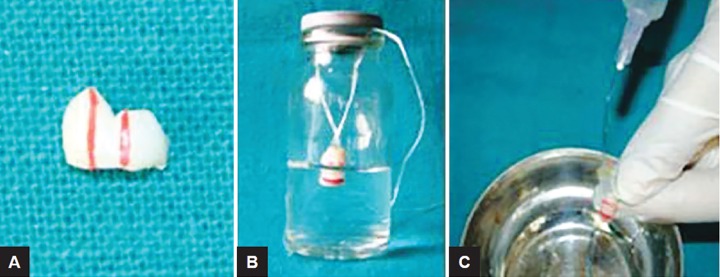
Demineralizing procedure (A) Sectioned tooth surface divided into three equal parts; (B) two-third of the tooth surface suspended in demineralizing solution using dental floss; and (C) rinsing with double-distilled water

**Figs 2A to C:**
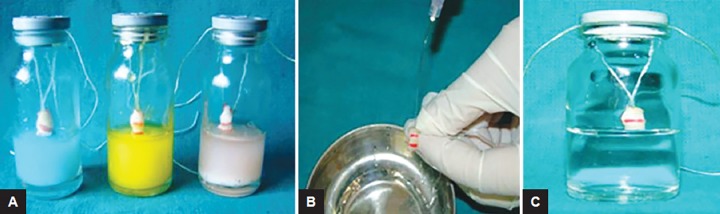
Remineralizing procedure (A) one-third of the tooth surface suspended in respective dentifrice slurry; (B) rinsing with double-distilled water; and (C) one-third of the tooth surface suspended in commercially available artificial saliva

### Remineralizing Procedure (Figs 2A to C)

Dentifrice slurry was freshly prepared every time during the study period by thoroughly mixing 1 gm of test dentifrice in 2.5 mL of double-distilled water and thoroughly centrifuging it at 4000 rpm for 20 minutes using a magnetic stirrer.

During remineralization cycle, lower one-third of each specimen was immersed in 5 mL of freshly prepared dentifrice slurry for 5 minutes, leaving the middle third demineralized surface as negative control. The specimens were then removed, washed with double-distilled water for 15 seconds, and placed in commercially available artificial saliva^¥^ for 8 hours to simulate oral environment.

Composition:

- Active ingredients-Each 5 mL contains Sodium Carboxymethyl cellulose 0.5%, Glycerine 30% in a pleasantly flavored base.
- Inactive ingredients: Sodium Saccharin, Sodium Methyl Paraben, Sodium Propylparaben, Lemon Oil.

After 8 hours, the specimens were removed from the artificial saliva, washed with double-distilled water and again treated with freshly prepared dentifrice solution for 5 minutes in the same manner as described earlier, followed by overnight placement of the specimen in artificial saliva. The procedure was repeated daily for 7 days, followed by examination of all the specimens using SEM.

### Scanning Electron Microscopy (Figs 3A to C)

Samples were rigidly mounted on a circular metallic sample holder with the help of sticky carbon tape. The samples were electrocoated with 20- to 50-nm thick gold using Bio-Rad Polaran sputter coater. After gold sputtering, the samples were subjected to SEM EVO 50. The scans were automatically generated on a computer attached to the SEM. An extremely high voltage [extra-high tension (EHT)] of 20 kV and magnification of 5000× was used to view sound, demineralized, and remineralized surface of all the 45 specimens.

The remineralization effect in all three test groups, positive, and negative controls was noted by an independent examiner according to the evaluation parameter (Table 1)^7^, which was then compared and evaluated statistically.

### Statistical Analysis

All the statistical was analyzed using SPSS software (version 15.0, Chicago, USA) and significance value was set at 0.05 or less. Descriptive statistics that included mean, mode, and standard deviation were calculated for each of the category. Categorical data were analyzed by ANOVA for differences between groups. For continuous data, paired t-test was performed.

## RESULTS

The three test groups were not statistically different from one another in terms of mean remineralization scores; although group A showed maximum remin-eralization (score = 3.27 ± 0.96), followed by group C (score = 2.87 ± 0.88) and B respectively (score = 2.73 ± 0.88) (Table 2). All the three test groups showed significant remineralization compared with demineralized enamel surface; however, the remineralization achieved was significantly low compared to the sound surface (Table 2).

## DISCUSSION

Due to the ailing consequences of dental caries, the focus of current research is shifting to development of new methodologies for the early detection and noninvasive treatment of carious lesions. Remineralization of early carious lesions is one of the major advancement in this field, which retards the progression of a lesion causing its arrest and achieves lesion regression ideally.^8^ Various means of remineralization became available, such as chewing gums (containing xylitol or tricalcium phosphate) and casein phosphopeptides-amorphous calcium phosphate (CPP-ACP) have been introduced over the time; however, their use in daily practice has not been established. Moreover, the risk of ingestion and hence, toxicity has been a cause of concern in pediatric patients. The same risks may be attributed to dentifrices when they are used both by adults and by children of the same family. Hence, pediatric dentifrices were marketed with claims of better tolerance and safety in pediatric patients. Due to low fluoride content of these dentifrices, it would be interesting to study their remineralization effect on the enamel; hence the present study was carried out comparing three commercially available pediatric dentifrices. Though the results were not statistically different from each other in the present study, group A showed a higher healing potential followed by group C and then by group B. The fluoride contents of three pediatric dentifrices used in our study were 1000 ppm sodium fluoride in group A (Dentifrice 1), 500 ppm sodium fluoride in group B (Dentifrice 2), and 498 ppm sodium monoflurophos-phate in group C (Dentifrice 3). The better result in group A could be attributed to higher fluoride content, although use of different fluoride agent could also be another reason. Also, group C contained xylitol, which acts on calcium ion carrier and maintains constant calcium ion content by introducing calcium ions from the surface layer to the middle and deep demineral-ized layers, thereby enhancing remineralization.^9,10^ The remineralizing potential of pediatric dentifrices has also been demonstrated in the earlier studies by Malekafzali and Tadayon,^11^ Itthagarun et al,^12^ and Ekambaran et al.^13^

**Figs 3A to C:**
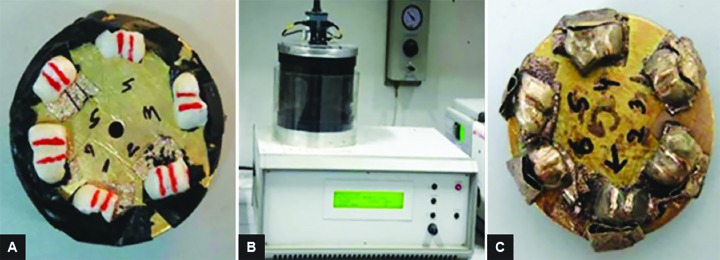
Scanning electron microscopy (A) Samples mounted on the metal stub; (B) gold sputtering unit; and (C) samples after gold sputtering

**Table 1:** **Table 1:** Evaluation parameters for remineralization

*Pattern observed*		*SEM photographs (present study)*		*Scoring criteria (Gupta et al 1998)^7^*	
Deep prismatic holes giving a typical honeycomb appearance and/or single or groups of focal holes.		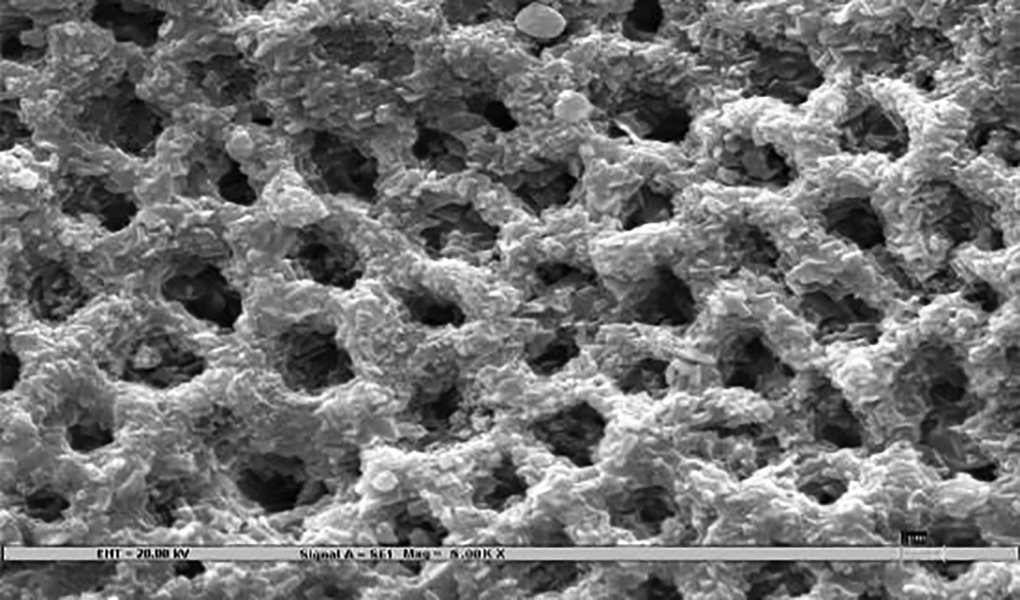		0 (Demineralized)	
Fine particle-like granular variable-sized deposits with uneven distribution.		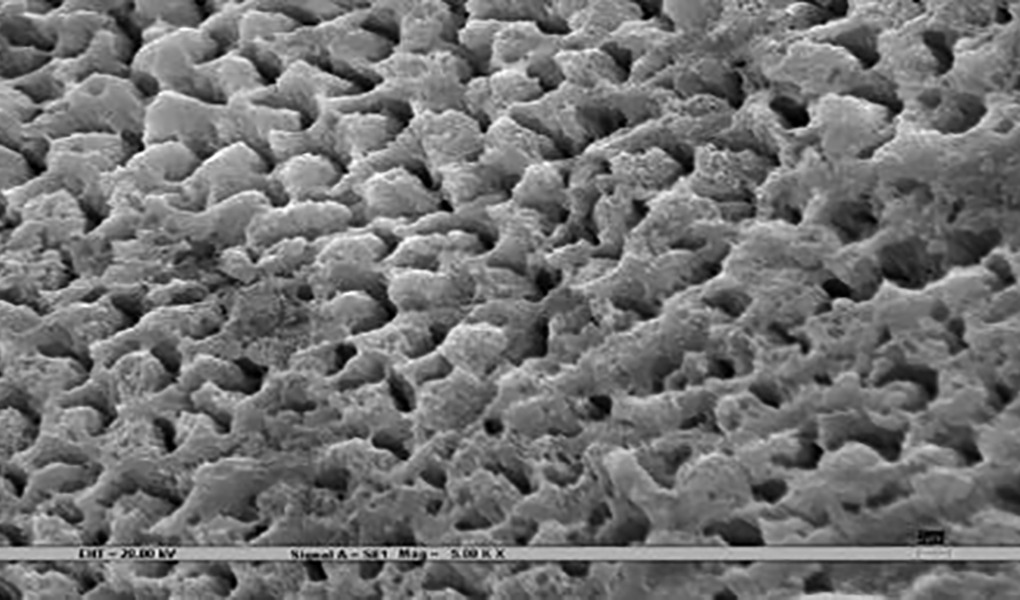		1	
Reduction in the depths of prismatic holes due to deposition of minerals within the prism cores. Honeycomb appearance still present.		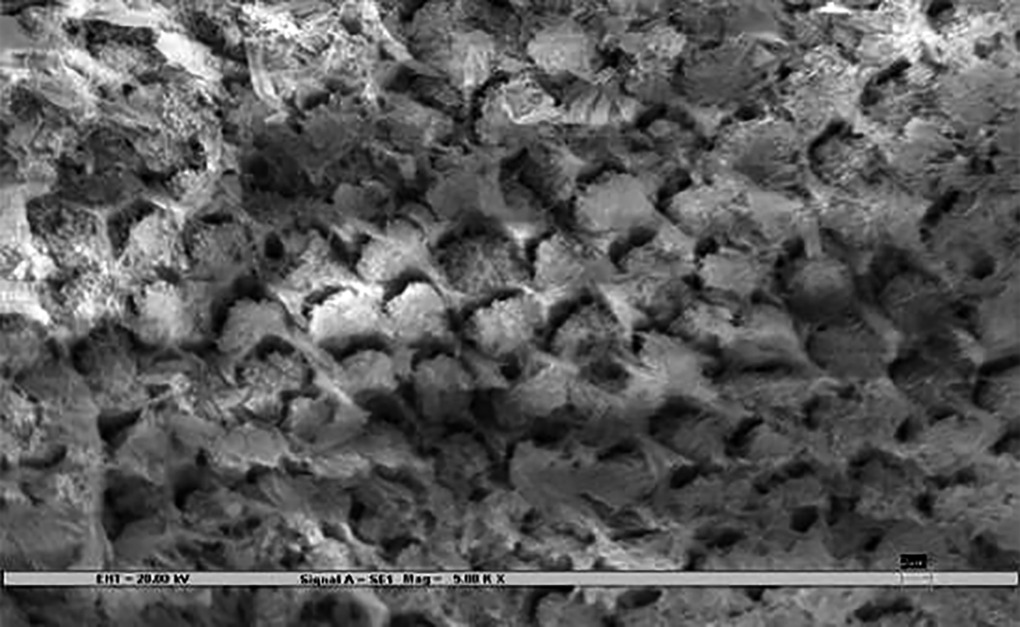		2	
Enamel prisms pits visible with mineral deposit only partially filling up prism cores. Honeycomb appearance not visible.		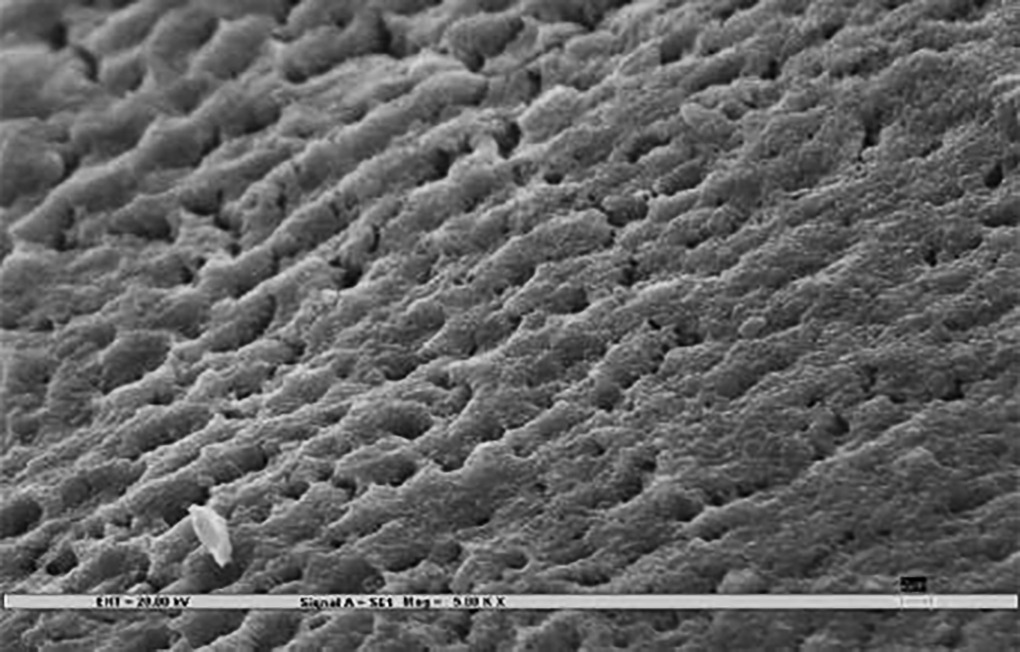		3	
Amorphous precipitate-like deposit scattered unevenly on the surface or globular particles deposited uniformly on the enamel surface.		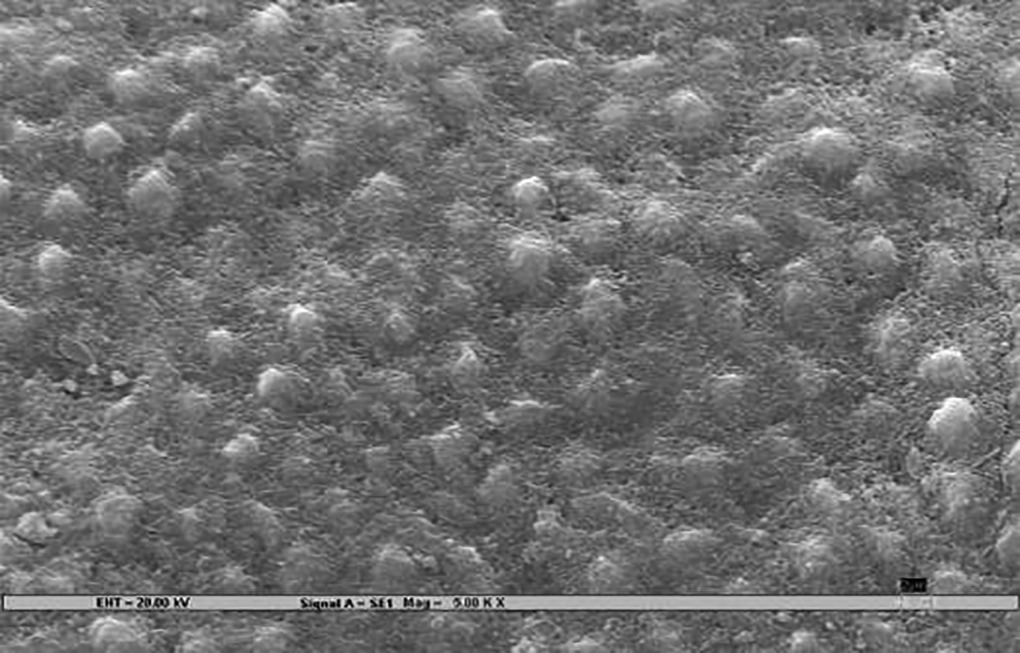		4	
Smooth homogeneous flat surface with slightly discernible prism shadows.		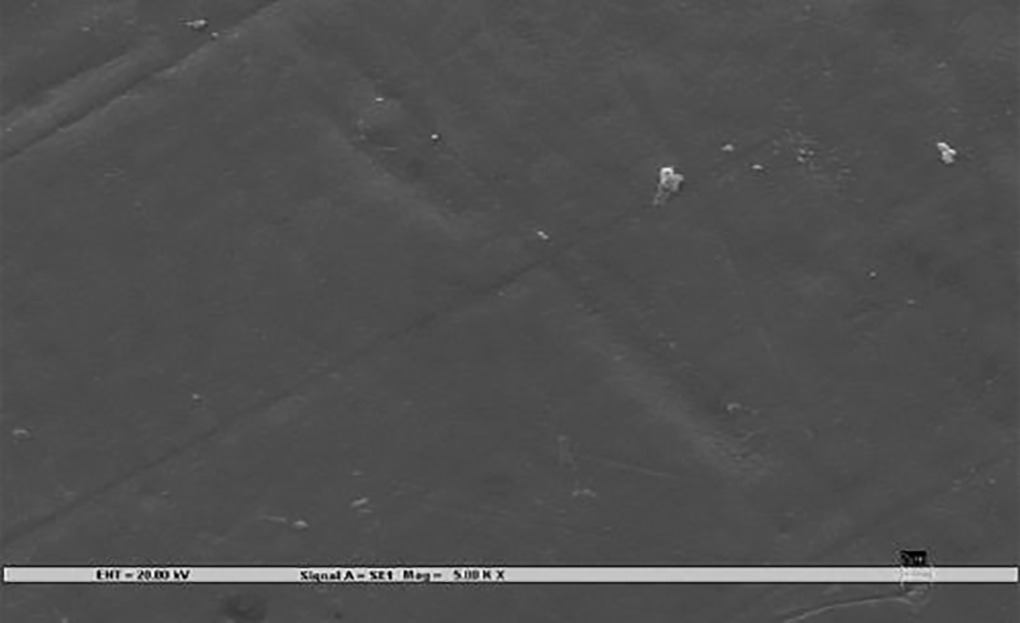		5 (Sound)	

**Table 2:** **Table 2:** Comparison between mean remineralization scores between demineralized and remineralized surface for all three groups

Groups		*Demineralized surface* *(mean score)*		*Sound surface* *(mean score)*		*Remineralized surface (mean score)*		*t-test*	
A		0^a^		5^b^		3.27 ± 0.96^c^		^a^ *^vs^* ^c^F value = -13.163; p < 0.001	
								^b^ *^vs^* ^c^F value = -6.985; p < 0.001	
B		0^a^		5^b^		2.73 ± 0.88^d^		^a^ *^vs^* ^d^F value=-11.979; p < 0.001	
								^b^ *^vs^* ^d^F value=-9.934; p < 0.001	
C		0^a^		5^b^		2.87 ± 0.91^e^		^a^ *^vs^* ^e^F value=-12.128; p < 0.001	
						^c^ *^vs^* ^d^ *^vs^* eANOVA=2.311 p = 0.267		^b^ *^vs^* ^e^F value=-9.025; p < 0.001	

a: demineralised surface score of 0; b: sound surface score of 5; c: remineralised surface mean score (Group A); d: remineralised surface mean score (Group B); e: remineralised surface mean score (Group C); Statistically significant at p < 0.001 [ANOVA test]

Experimental model based on the formation of lesions in *in vitro* systems can be used to understand the effects of such agents on carious processes. However, *in vitro* systems face criticism because of the mechanical limitations in various ways:^14,15^ (1) They are unable to completely simulate the complex intraoral conditions leading to caries development, even when “artificial mouth” systems, bacterial biofilms, and saliva are employed. This is particularly relevant for testing fluoridated dentifrices with monofluorophosphate (MFP), since the enzyme systems required for MFP hydrolysis are present in saliva and plaque *in vivo,* but are absent in most *in vitro* test methods; (2) they cannot mimic solid surface area/solution ratios or the saliva/ plaque fluid composition encountered *in vivo,* since different oral surfaces are bathed in different volumes and source combinations of saliva, (3) there are artifacts associated with the choice of substrate and test conditions, particularly the time periods of de- and remineralization, which are much faster than those expected to occur in *in vivo* conditions; and (4) they are not able to adequately simulate topical use and clearance of products from the oral cavity. While dentifrices are typically slurried to simulate dilution during brushing, the uptake and reactivity of fluoride are consistently lower *in vivo* than *in vitro,* which may result in inaccurate assessments of the anticaries potential of formulations directed toward enhancement of fluoride delivery. All these limitations must be kept in mind when data from pH cycling studies are intended to be extrapolated for the clinical situations. In the oral cavity, the pH alterations are more frequent depending on individual’s dietary and oral hygiene habits; therefore, it is difficult to exactly simulate the oral conditions that prevail in the mouth. Nevertheless, there is greater control over these variables in an *in vitro* model, which may be difficult to obtain in a mouth. The present study tried to simulate oral conditions as far as possible, viz., 5 minutes’ suspension in dentifrice slurry every 8th hour (simulating 5 minutes of brushing twice daily) and suspension in artificial saliva (simulating the effect of saliva in oral cavity). Various methods have been used by different authors to demineralize enamel^16,17^; however, the present method was modified from ten Cate and Duijsters^6^ because of the convenience in the reduced time period of immersion and easy availability of chemicals. Time period of 96 hours was used for demineralization based on the study by Rirattanapong et al^18^ to produce 60 to 100 μm deep artificial carious lesions. Various methods have been used for evaluating the remineralization of white spot lesions, such as clinical evaluation, polarized microscopy, energy dispersion X-ray analysis (EDX), microhardness test, chemical analysis, stereomicroscopy, and SEM. The present study employed SEM with high-resolving power because of reported high sensitivity toward early reactions occurring at crystal level.^1^

The objective of any fluoride preventive therapy is to attain maximum anticaries action with the minimal risk of fluorosis. This risk is a function of both the amount of dentifrice and the fluoride concentration. Fluoride dentifrices may account for 57% of the total fluoride ingested in 4- to 6-year age group children due to their inability to spit out the toothpaste during brushing.^19^ These concerns have led to recommendations to minimize fluoride ingestion during tooth-brushing by using a small amount of toothpaste by children and incorporating minimal quantity of fluoride in the toothpastes. Hence, from the present study, it can be concluded that all three dentifrices tested showed remineralizing potential although insignificantly different from each other but significantly higher compared to the demineralizing surface. Future studies are needed utilizing pH cycling method to simulate oral environment and testing the remineralization potential using microhardness test.

## CONCLUSION

From the study, it can be concluded that all the three pediatric dentifrices showed remineralizing potential, which was significantly high compared to the control demineralized surface but low compared to the sound surface. Group A showed better remineralizing potential, followed by groups C and B respectively, possibly due to different anticariogenic constituents. Thus, one can use pediatric dentifrices for preventing dental caries and decelerating lesion progression with an added advantage of lower fluoride toxicity risk.
